# β-elemene Isopropanolamine Derivative LXX-8250 Induces Apoptosis Through Impairing Autophagic Flux *via* PFKFB4 Repression in Melanoma Cells

**DOI:** 10.3389/fphar.2022.900973

**Published:** 2022-08-10

**Authors:** Sajid Jalal, Ting Zhang, Jia Deng, Jie Wang, Ting Xu, Tianhua Zhang, Chuanxin Zhai, Ruqiang Yuan, Hongming Teng, Lin Huang

**Affiliations:** ^1^ Department of Pathophysiology, College of Basic Medical Sciences, Dalian Medical University, Dalian, China; ^2^ Liaoning Provincial Key Laboratory of Medical Molecular Biology, Dalian, China; ^3^ Liaoning Key Laboratory of Molecular Recognition and Imaging, School of Bioengineering, Dalian University of Technology, Dalian, China; ^4^ Advanced Institute for Medical Sciences, Dalian Medical University, Dalian, China

**Keywords:** autophagy, glycolysis, antitumor effects, melanoma, apoptosis

## Abstract

Melanoma is a highly aggressive skin cancer and accounts for most of the skin cancer-related deaths. The efficacy of current therapies for melanoma remains to be improved. The isopropanolamine derivative of β-elemene LXX-8250 was reported to present better water solubility and stronger toxicity to tumor cells than β-elemene. Herein, LXX-8250 treatment showed 4-5-fold more toxicity to melanoma cells than the well-known anti-melanoma drug, Dacarbazine. LXX-8250 treatment induced apoptosis remarkably, which was caused by the impairment of autophagic flux. To clarify the molecular mechanism, microarray analyses were conducted, and PFKFB4 expression was found to be suppressed by LXX-8250 treatment. The cells overexpressed with PFKFB4 exhibited resistance to apoptosis induction and autophagic flux inhibition by LXX-8250 treatment. Moreover, LXX-8250 treatment suppressed glycolysis, to which the cells overexpressed with PFKFB4 were tolerant. LXX-8250 treatment inhibited the growth of melanoma xenografts and suppressed PFKFB4 expression and glycolysis *in vivo*. Taken together, LXX-8250 treatment induced apoptosis through inhibiting autophagic flux and glycolysis in melanoma cells, which was mediated by suppression of PFKFB4 expression. The study provides a novel strategy to melanoma treatment.

## Introduction

Cancer is one of the most life-threatening diseases. The estimated incidence of all cancer types worldwide in 2020 is 247.5 per 100,000, and an estimated 49% increase in new cases and a 62% increase in deaths will occur in 2040 compared with 2020 (Cao et al., 2021). Melanoma is a highly aggressive skin cancer, accounting for the 75% skin cancer related death (Cullen et al., 2020; Siegel et al., 2020). Estimated new cases of melanoma for 2022 account for 5% in females and 6% in males among all new diagnoses of cancer (Siegel et al., 2022). The worldwide incidence of melanoma increases 3% annually (Tripp et al., 2016). Melanoma metastasizes early, and is resistant to treatment especially in later stage, thus poor therapeutic responses often occur (Rodriguez-Hernandez et al., 2020; Shi et al., 2020). Therefore, it is urgent to develop a novel approach to melanoma treatment.

β-elemene (1-methyl-1-vinyl-2, 4-diisopropenyl-cyclohexane) is a sesquiterpene compound extracted from natural plants, such as *Curcuma aromatica*, *Curcuma zedoaria* and *Curcuma wenyujin*, and has been used against many kinds of tumors (Wu et al., 2017; Wu et al., 2019; Liu et al., 2020; Zhang et al., 2020). Although there are researches on β-elemene treatment in melanoma cell lines and mouse models, no clinical attempt has been performed (Chen et al., 2011; Shi et al., 2014; Shi et al., 2015). Moreover, poor water solubility and moderate activity limit the clinical use of β-elemene. LXX-8250 {3,3′-[Piperazine-1,4-diyl]-bis[1-2-(1R,3S,4S)-4-methyl-3-(prop-1-en-2-yl)-4-vinylcyclohexyl allyl oxypropan-2-ol]} is a novel isopropanolamine derivative of β-elemene, with higher water solubility and stronger toxicity to tumor cells compared to β-elemene and other derivatives (Chen et al., 2017). Therefore LXX-8250 might be an ideal alternative of β-elemene. However, the detailed mechanism of LXX-8250’s antitumor effects remains unknown. In addition, no research has been conducted on the availability of LXX-8250 treatment for melanoma to date yet.

Warburg effect occurs in most tumors and tumor cells prefer to generate energy through glycolysis. In the melanoma cells resistant to BRAF inhibitors, glycolysis inhibition was shown to be able to induce cell death (Parmenter et al., 2014; Paluncic et al., 2016). PFKFB (6-phosphofructo-2-kinase/fructose-2,6-bisphosphatase) is a family of 4 bi-functional and rate-limiting isoenzymes of glycolysis, catalyzing both the synthesis and degradation of fructose-2,6-biphosphate (F-2,6-BP) which activates PFK-1 to increase glycolysis flux to produce lactic acid (Kotowski et al., 2021). Overexpression of PFKFB4 has been documented in plenty of tumors, including breast cancer, renal clear cell carcinoma, etc. (Goidts et al., 2012; Ros et al., 2012; Gao et al., 2018; Feng et al., 2021). PFKFB4 contributes to tumorigenesis by mediating glycolysis in many kinds of tumors (Ros et al., 2017; Dasgupta et al., 2018; Feng et al., 2021). These observations suggest that PFKFB4 could be a potential therapeutic target for tumor treatment.

Glycolysis has been shown to promote tumor progression by regulating autophagy (Marcucci and Rumio, 2022). Autophagy functions as a double-edged sword to facilitate cell proliferation or stimulate cell death dependent on different cellular context (Denton and Kumar, 2019; Klionsky et al., 2021). Glycolytic enzymes including PGK1, PKM2, LDHA, etc. are involved in the induction of the autophagy which promotes tumor proliferation (Qian et al., 2017; Das et al., 2019; Wang et al., 2019; Ikeda et al., 2020). However, in some cases, glycolysis inhibits the autophagy mediating cell death (Pollard et al., 2014; Wang et al., 2016; Zhang et al., 2021a). One of the mechanisms through which PFKFB4 facilitates the proliferation and chemoresistance of tumor cells is controlling autophagy (Strohecker et al., 2015; Wang et al., 2018a). PFKFB4 knockdown significantly impairs autophagic flux in prostate cancer cells (Strohecker et al., 2015), while suppresses autophagy induction in SCLC cells (Wang et al., 2018a). Nevertheless PFKFB4-mediated glycolysis contributes to the autophagy regulation in cancer.

Herein we report that LXX-8250 treatment induces apoptosis in melanoma cells *via* suppressing autophagic flux. Furthermore, LXX-8250 is determined as a novel inhibitor of PFKFB4 and glycolysis, and the suppression of PFKFB4 expression is found to be able to mediate the antitumor effects of LXX-8250. This study will provide a novel insight for the development of melanoma therapy and the understanding of LXX-8250’s antitumor effects and mechanism.

## Materials and Methods

### Cell Lines and Cell Cultures

Human melanoma cell lines A2058 and SK-MEL-28, and Ewing’s sarcoma cell line A673 were obtained from ATCC. HEK293T, CCC-HEL-1, HL-7702, and Mel JuSo were tested for eight STR loci and amegloenin gene. All cell lines were tested negative for mycoplasma. HL-7702 cells were cultured in RPMI medium and the other cells were cultured in DMEM medium supplemented with 10% FBS at 37°C with 5% CO_2_ in a humidified atmosphere for less than 6 months after resuscitation.

### Antibodies and Chemicals

Antibodies were bought for the detection of PFKFB4 (GeneTex); LC3, PARP (Cell Signaling Technology); β-Actin (Proteintech) and P62 (Abcam). LXX-8250 (95%) was chemically synthesized and obtained from Yuanda Pharmaceuticals (Dalian, China). Z-VAD and 3-MA were from MedChemExpress. Chloroquine diphosphate (CQ) was from Sigma-Aldrich and Dacarbazine was from Topscience. Lactic acid detection kit was from Nanjing Jiancheng Bioengineering Institute, and fructose-1,6-biphosphate (F-1,6-BP) detection kit was from Suzhou Grace Biotechnology.

### Plasmid Construction

shRNA sequence against PFKFB4 was designed as 5′-GAC​GTG​GTC​AAG​ACC​TAC​AAA-3′. Oligos were annealed and ligated with AgeI - EcoRI sites into the pLKO.1 vector to construct shRNA for PFKFB4. FLAG-PFKFB4 was constructed by inserting the full length of human PFKFB4 cDNA with a FLAG sequence at the N-terminus into EcoRI—SpeI sites of pSin-EF2 vector.

### Lentivirus Production and Transduction

Lentiviruses were harvested 48 h after expression vector transfection with packaging plasmid psPAX2 (a gift from Dr. Didier Trono, Addgene plasmid #12260) and envelope plasmid pMD2.G (a gift from Dr. Didier Trono, Addgene plasmid #12259) into HEK293T cells using Lipofectamine 2000 reagent (Thermo fisher). Cells were infected with lentivirus at the presence of 8 μg/ml Polybrene (Sigma), prior to 2 μg/ml puromycin selection for 72 h to establish stable cell lines.

### Cell Viability Assay

5 × 10^3^ cells/well were seeded in triplicate into 96-well plate. After 24 h, LXX-8250 was added at the indicated concentration and cell viability was determined with Cell Counting Kit-8 (Dojindo, Kumamoto, Japan) 24 h or 48 h later. Absorbance at 450 nm with the reference at 630 nm was measured. Cell viability was calculated as:

Cell viability (%)=(ODsample−ODblank)/(ODcontrol−ODblank)×100

#### Colony Formation Assay

Cells were seeded at 1,000/well into 6-well plates and treated with various concentrations of LXX-8250. After 24 h, cells were washed with PBS and supplemented with fresh media. Fourteen days later, the colonies formed were stained with 0.1% crystal violet and the colonies with a diameter >1 mm were counted.

### Apoptosis Assay

Apoptosis was measured with a fluorescence-activated cell sorter Accuri C6 (BD) by using Annexin V, FITC Apoptosis Detection Kit (Dojindo, Kumamoto, Japan). Cells were collected after 24 h-treatment, followed with staining by FITC-labeled annexin V and PI.

### Monitoring Autophagy Flux

mRFP-EGFP-LC3 transfected cells were treated with LXX-8250 or CQ (10 µM) for 24 h. Images were taken by a confocal microscope (Leica TCS SP5×). RFP^+^EGFP^+^ LC3 puncta (yellow dots) were deemed as autophagosomes, while RFP^+^EGFP^−^ LC3 puncta (red dots) were deemed as autolysosome (Mizushima et al., 2010; Klionsky et al., 2021).

### Microarray Analysis

A2058 cells were treated with LXX-8250 (14.6 μM) or DMSO for 8 h. Total RNA of 3 replicates for each treatment was isolated with TRI reagent (Sigma). Gene microarray analyses were performed by Phalanx Biotech Group using the Human OneArray^®^ V7.1 microarray. The chips were scanned with Agilent Microarray Scanner G2505C (Agilent), and the results were normalized and log-transformed with Agilent 0.1 XDR software (Agilent).

### Lactic Acid Assay

Cells were cultured in FBS free medium for 24 h, and the media were collected and applied to the lactic acid assay kit following the manufacturer’s instructions. The absorbance was measured at 530 nm, and the lactic acid concentration was calculated as the following formulation: Lactic acid concentration  (mM)=(testOD−blankOD)/(standardOD−blankOD)×standard sample’ s concentration(3mM) .

### Fructose-1,6-Biphosphate Assay

Cells were cultured in FBS free medium for 24 h, and the media were collected and applied to the F-1,6-BP assay kit following the manufacturer’s instructions. The absorbance was measured at 340nm, and the concentration was calculated. Relative amount of F-1,6-BP in culture media was presented.

### *In Vivo* Tumorigenicity Assay

All animal maintenance and procedures were performed according to the recommendations of the Animal Care and Ethics Committee of Dalian Medical University. The protocol was approved by the Animal Care and Ethics Committee of Dalian Medical University (January 2022/No. AEE22054). All efforts were made to minimize mice suffering.

Mice were maintained and mice experiments were conducted in the specific-pathogen-free (SPF) Laboratory Animal Center of Dalian Medical University. A2058 cells (5×10^6^) were subcutaneously injected into the posterior flanks of male BALB/c nude mice (Vital River, Beijing). When the xenograft tumors formed were detectable by palpation, the injected mice were randomly divided into control and LXX-8250 treated groups. From day 4, LXX-8250 (20 mg/kg) was started to be injected in peritumoral region once a day for up to 16 days (*n* = 6/group). Every 2 days, tumor volume was measured with a caliper, and calculated by the formula V = 1/2 (width^2^×length). Body weights were also obtained. On day 17, all the mice were sacrificed after anaesthesia with pentobarbital (45 mg/kg), and the tumors formed were dissected, weighed and measured.

### Statistical Analysis

For microarray analyses, data were analyzed by Rosetta Resolver 7.2 with error model. For the other experiments, differences between groups were assessed by a two-sided Student’s t-test using GraphPad Prism 7.04 software. All the experiments were performed in 3 independent experimental replications. Data were presented as mean ± standard deviation (SD). *p* < 0.05 was considered statistically significant.

## Results

### LXX-8250 Treatment Induces Apoptosis in Melanoma Cells

**FIGURE 1:**
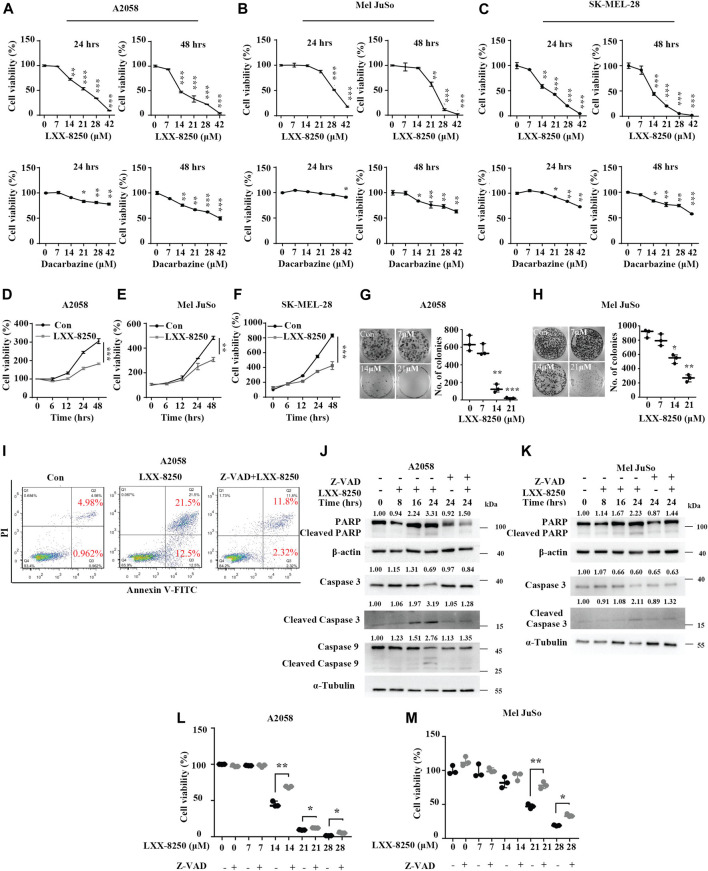
Effects of LXX-8250 on melanoma cells. **(A–F)** Cell viability assays in A2058 **(A,D)**, Mel JuSo **(B,E)** or SK-MEL-28 **(C,F)** cells. Cells were treated with increasing concentration of LXX-8250 or Dacarbazine for the indicated time **(A–C)**, or at 14.6 µΜ **(D)**, 22.8 µΜ **(E)** or 13.5 µΜ **(F)** respectively for increasing time. **(G,H)** Colony formation assays in A2058 **(G)** or Mel JuSo **(H)** cells treated with LXX-8250 at the indicated concentration. **(I)** Apoptosis assays in the cells treated without (Con) or with LXX-8250 (14.6 µΜ) alone or LXX-8250 (14.6 µΜ) together with Z-VAD (50 µΜ). **(J,K)** Western blot analysis for the cells treated with LXX-8250 14.6 µΜ, **(J)**; 22.8 µΜ, **(K)** or LXX-8250 14.6 µΜ, **(J)**; 22.8 µΜ, **(K)** together with Z-VAD (50 µΜ) at the indicated time. The quantification results of the band density (Cleaved PARP, Caspase 3, Cleaved Caspase 3, and Cleaved Caspase 9) were labeled above the bands. **(L,M)** Cell viability assays in the cells treated with increasing concentration of LXX-8250 together with or without Z-VAD (50 µΜ). Data represent mean ± SD. *p < 0.05, **p < 0.01, ***p < 0.001.

LXX-8250 (Supplementary Figure S1A) showed less toxicity to normal cells than a well-known anti-melanoma drug, Dacarbazine (Supplementary Figures S1B,C). To investigate the effects of LXX-8250 on melanoma cells, we treated cells with increasing concentrations of LXX-8250 or Dacarbazine, and analyzed the cell viability. The cells showed more remarkable dose-dependent decline in the cell viability of both 24 h- and 48 h-LXX-8250-treated cells than Dacarbazine-treated cells. IC_50_ at 48 h of LXX-8250 treatment is 14.6 µΜ, 22.8 µΜ or 13.5 µΜ, while that of Dacarbazine treatment is 83.93 µΜ, 94.17 µΜ or 76.02 µΜ for A2058, Mel JuSo or SK-MEL-28 cells, respectively (*p* < 0.01, Figures 1A–C, Supplementary Figure S1D). A similar reduction in viability of LXX-8250-treated Ewing’s sarcoma cell A673 was also observed (*p* < 0.01, Supplementary Figure S1E). Next, the concentration of 14.6 µΜ, 22.8 µΜ or 13.5 µΜ was used against A2058, Mel JuSo or SK-MEL-28 cells, and all the cell lines displayed significant decreases in cell viability with LXX-8250 treatment in a time-dependent manner (*p* < 0.01, Figures 1D–F). LXX-8250 treatment also suppressed colony formation dose dependently, consistent with the results of cell viability assays (Figures 1G,H).

Furthermore, we found that LXX-8250 treatment led to cell death (Supplementary Figures S1F,G). To elucidate whether LXX-8250 treatment induces apoptosis, we performed apoptosis assays. Cells were treated with LXX-8250 and stained with PI/Annexin V. The flow cytometry analyses showed that the ratio of PI־/Annexin V⁺ cells representing early apoptosis was increased to 12.5% from 0.962%, which was restored to 2.32% by the combination with an apoptosis inhibitor Z-VAD. While the ratio of PI⁺/Annexin V⁺ cells representing late apoptosis was increased to 21.5% by LXX-8250 treatment from 4.98%, which was also restored to 11.8% by the combination with Z-VAD (Figure 1I). Moreover, the levels of apoptosis markers, cleaved poly ADP-ribose polymerase (PARP), cleaved caspase 3 and cleaved caspase 9, were all augmented by LXX-8250 treatment, which was reversed in the cells treated with the combination of LXX-8250 and Z-VAD (Figures 1J,K). Additionally, the results of cell viability assays showed that Z-VAD treatment partially restored the viability repressed by LXX-8250 treatment (*p* < 0.01, Figures 1L,M). Thus LXX-8250 treatment induced apoptosis of melanoma cells.

### LXX-8250 Treatment Suppresses Autophagic Flux to Promote Apoptosis

**FIGURE 2:**
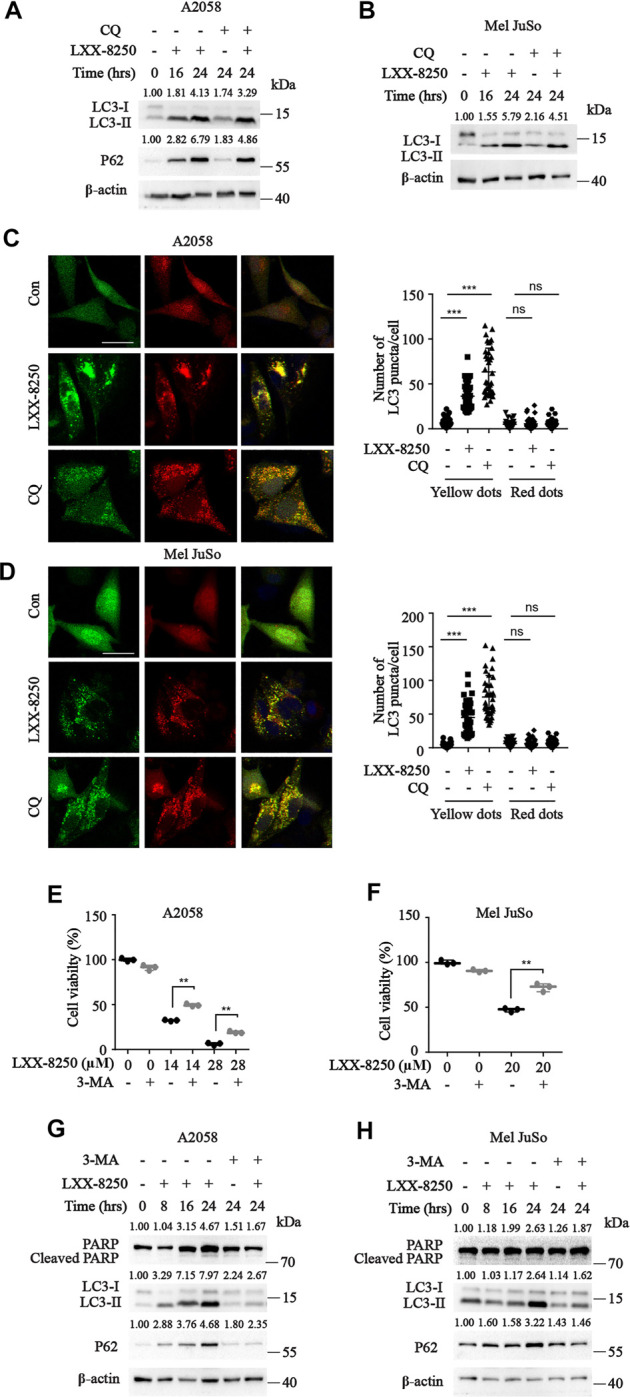
Effects of LXX-8250 on autophagy. **(A,B)** Western blot analysis for the cells with the indicated treatment at the indicated time. LXX-8250 14.6 µΜ, **(A)**; 22.8 µΜ, **(B)**; CQ, 10 μM. **(C,D)** The indicated cells transfected with mRFP-EGFP-LC3 were treated with LXX-8250 14.6 µΜ, **(C)**; 22.8 µΜ, **(D)** or CQ (10 μM) for 24 h. LC3 puncta were shown in representative micrographs with a scale bar of 25 μm. Thirty-six cells of each treatment were counted for puncta, and the numbers (autophagosomes, yellow dots; autolysosome, red dots) per cell were plotted. **(E,F)** Cell viability assays in the cells treated with the indicated concentration of LXX-8250 together with or without 3-MA (2 mM) for 48 h **(G,H)** Western blot analysis for the cells treated with LXX-8250 14.6 μM, **(G)**; 22.8 µΜ, **(H)** for the indicated times together with or without 3-MA (2 mM). The quantification results of the band density (LC3-II, P62 and Cleaved PARP) were labeled above the bands. Data represent mean ± SD. **p < 0.01, ***p < 0.001.

In order to explore the mechanism of the apoptosis induced by LXX-8250 treatment, we considered the possibility of autophagy regulation. After LXX-8250 treatment, significant increases in LC3-II expression levels were observed. Simultaneously the expression levels of P62, one of the substrates of autophagy, were also upregulated remarkably. To verify the autophagy process affected, we next treated the cells with a lysosomal inhibitor chloroquine diphosphate (CQ) to impair autophagosome fusion with lysosome. There was not any change in LC3-II levels between the cells treated with CQ along with LXX-8250 and those treated with LXX-8250 only (Figures 2A,B). Thus, lysosomal inhibition could not enhance LC3-II accumulation caused by LXX-8250 further. Further ULK1 phosphorylation and Beclin 1 expression, which are generally involved in autophagy induction, were not changed by LXX-8250 treament (Supplementary Figure S2). Moreover, we examined LC3 puncta formation in mRFP-EGFP-LC3 introduced cells. LXX-8250 treatment increased the average number of RFP⁺EGFP⁺ LC3 puncta (autophagosome, yellow dots) from 6.6 to 36.2 per cell in A2058 cells and from 4.4 to 44.6 per cell in Mel JuSo cells respectively, whereas the average number of RFP⁺EGFP־LC3 puncta (autolysosome, red dots) was not changed at all. These results are similar to the phenomena shown in CQ treated cells (*p* < 0.001, Figures 2C,D), which indicates that the effects of LXX-8250 treatment on autophagy are mainly caused by blocking the ligation of autophagosome to lysosome instead of promoting autophagosome formation. Therefore LXX-8250 treatment increased autophagosome accumulation through impairing autophagic flux in melanoma cells.

To clarify whether the inhibition of autophagic flux by LXX-8250 treatment results in apoptosis, we used 3-MA, an autophagy inhibitor, to treat cells along with LXX-8250. As the results, 3-MA treatment downregulated the levels of LC3II and P62 induced by LXX-8250 treatment, and restored the cell viability of LXX-8250-treated cells significantly (*p* < 0.01, Figures 2E,F). Further the levels of cleaved PARP increased by LXX-8250 treatment were partially restored by 3-MA treatment (Figures 2G,H). These results suggest that LXX-8250 treatment leads to apoptosis through upregulation of autophagosome accumulation.

### LXX-8250 Treatment Downregulates PFKFB4 Expression in Melanoma Cells

**FIGURE 3:**
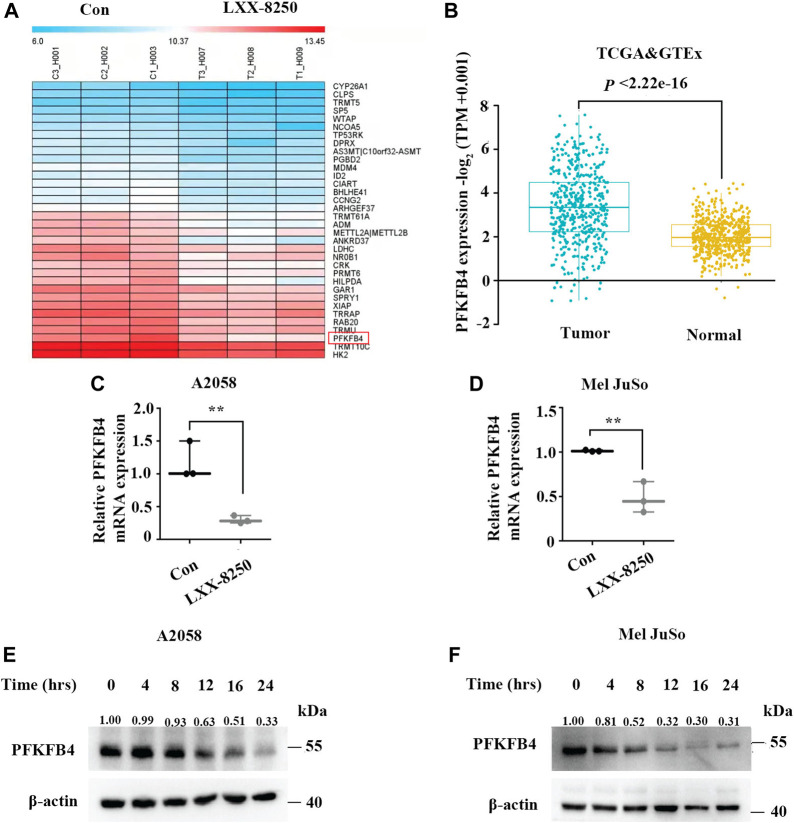
Effects of LXX-8250 on PFKFB4 expression. **(A)** Heatmap for the representative downregulated genes in LXX-8250 (14.6 μM) treated cells compared with control cells, as evaluated by microarray analyses (fold change>1.5, *p* < 0.05). **(B)** The relative PFKFB4 mRNA expression levels in SKCM data from TCGA combined with Skin data from GTEx data. Tumor, 469 cases; Normal, 557 cases. **(C,D)** Real time PCR analyses for the cells treated with LXX-8250 14.6 μM, **(C)**; 22.8 µΜ, **(D)**. **(E,F)** Western blot analysis for the cells treated with LXX-8250 14.6 μM, **(E)**; 22.8 µΜ, **(F)** for the indicated times. The quantification results of the band density were labeled above the bands. Data represent mean ± SD. **p < 0.01, ***p < 0.001.

To investigate further the mechanism of LXX-8250’s antitumor effects, A2058 cells were treated with LXX-8250, followed by microarray analyses. The results revealed that 34 genes were downregulated in LXX-8250 treated cells (fold change >1.5, *p* < 0.05, Figure 3A), among which PFKFB4 expression was decreased by 2.56 folds and ranked to No. 2 dependent on the fold change. Further, the relative mRNA expression of PFKFB4 in SKCM (Skin Cutaneous Melanoma) data from TCGA (The Cancer Genome Atlas) combined with Skin data from GTEx (Genotype-Tissue Expression) was significantly higher (*p* < 2.22e-16) in tumor (469 samples) than that of normal (557 samples) (Figure 3B). Thus, we decided to focus on the role of PFKFB4 in LXX-8250’s effects. To validate the results of microarray analyses, we performed real time PCR analysis. The cells treated with LXX-8250 showed a significant decrease in PFKFB4 mRNA expression levels than those in control cells (*p* < 0.001, Figures 3C,D). Similarly, PFKFB4 protein levels were decreased remarkably by LXX-8250 treatment time-dependently (Figures 3E,F). These results suggest that LXX-8250 treatment suppresses PFKFB4 expression in melanoma cells.

### LXX-8250’s Effects are Mediated Through PFKFB4 Repression

**FIGURE 4:**
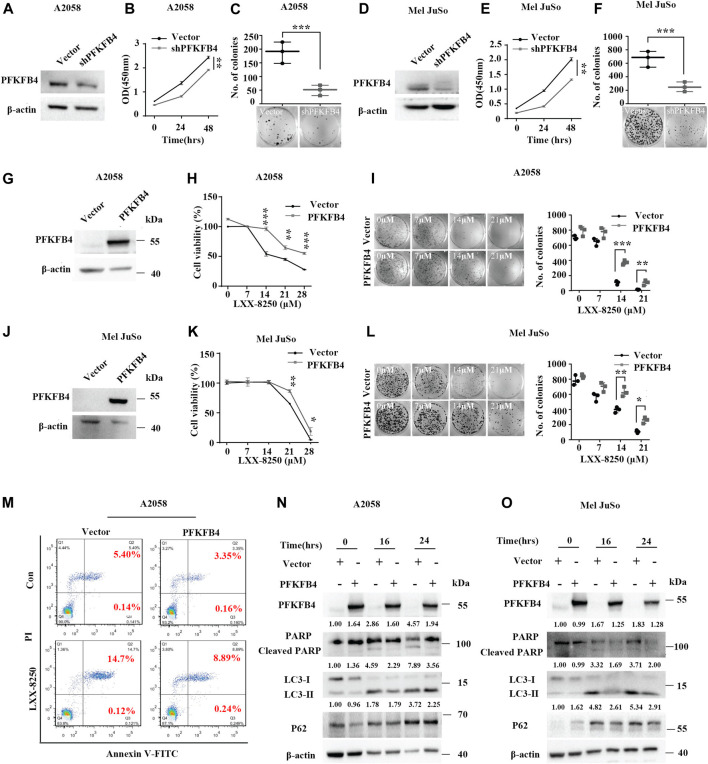
Effects of PFKFB4 on LXX-8250 induced apoptosis. **(A–F)** Empty vector or shRNA (shPFKFB4) was introduced into A2058 or Mel JuSo cells, followed with Western blot analysis **(A,D)**, cell viability assays **(B,E)** and colony formation assays **(C,F)**. **(G–L)** The A2058 or Mel JuSo cells introduced with empty vector or PFKFB4 overexpression plasmid were treated with LXX-8250 at the indicated concentration for 48 h, followed with Western blot analysis **(G,J)**, cell viability assays **(H,K)** and colony formation assays **(I,L)**. **(M)** The cells introduced with empty vector or PFKFB4 overexpression plasmid were treated with or without LXX-8250 (14.6 μM), followed by apoptosis assays. **(N,O)** The cells introduced with empty vector or PFKFB4 overexpression plasmid were treated with LXX-8250 14.6 μM, **(N)**; 22.8 µΜ, **(O)** for the indicated time, followed by Western blotting analysis. The quantification results of the band density (LC3-II, P62 and Cleaved PARP) were labeled above the bands. Data represent mean ± SD. *p < 0.05, **p < 0.01, ***p < 0.001.

To explore the role of PFKFB4 further in LXX-8250’s effects, first we examined the influence of PFKFB4 on the cell proliferation of melanoma cells. We knocked down PFKFB4 with shRNA transfection (shPFKFB4) in A2058 cells and checked the cell viability. shPFKFB4 transfection significantly repressed cell viability compared with empty vector transfection (*p* < 0.01, Figures 4A,B). Also, the number of colonies formed in shPFKFB4 transfected cells were less than those in empty vector transfected cells (*p* < 0.001, Figure 4C). Similar results were obtained in Mel JuSo cells (Figures 4D–F).

Next, LXX-8250 treatment showed a much less toxicity to the cells with PFKFB4 overexpression than the cells introduced with empty vector (*p* < 0.001–0.05, Figures 4G–L). To investigate the effects of PFKFB4 on the apoptosis induced by LXX-8250 treatment, we conducted the apoptosis assays in PFKFB4-overexpressing cells. The rate of PI⁺/Annexin V⁺ cells representing late apoptosis in the empty vector-transfected cells was increased to 14.70% by LXX-8250 treatment while that in the PFKFB4-transfected cells was only increased to 8.89% (Figure 4M). The levels of cleaved PARP were significantly upregulated in shPFKB4 knockdown cells, while downregulated in PFKFB4-overexpressing cells (Supplementary Figure S3). Moreover, although LXX-8250 treatment raised the cleaved PARP levels significantly in control cells, it nearly failed to change the cleaved PARP levels in PFKFB4-overexpressing cells (Figures 4N,O). These results suggested that the PFKFB4-overexpressing cells were resistant to the toxicity of LXX-8250 treatment. Therefore LXX-8250 treatment may induce apoptosis *via* suppressing PFKFB4 expression.

In addition, LC3-II expression was increased in PFKFB4-knockdown cells while decreased in PFKFB4-overexpressing cells. The same was true for P62 levels (Supplementary Figure S3). Consistently, although LXX-8250 treatment upregulated LC3-II levels notably in control cells, the upregulation of LC3-II levels in PFKFB4-overexpressing cells were much weaker. The same was also true for P62 levels (Figures 4N,O). These data indicated that PFKFB4-overexpressing cells were resistant to LXX-8250-impaired autophagic flux. Thus LXX-8250 treatment inhibits autophagic flux to facilitate apoptosis by repressing PFKFB4 expression.

### LXX-8250 Treatment Inhibits Glycolysis *via* PFKFB4 Repression

**FIGURE 5:**
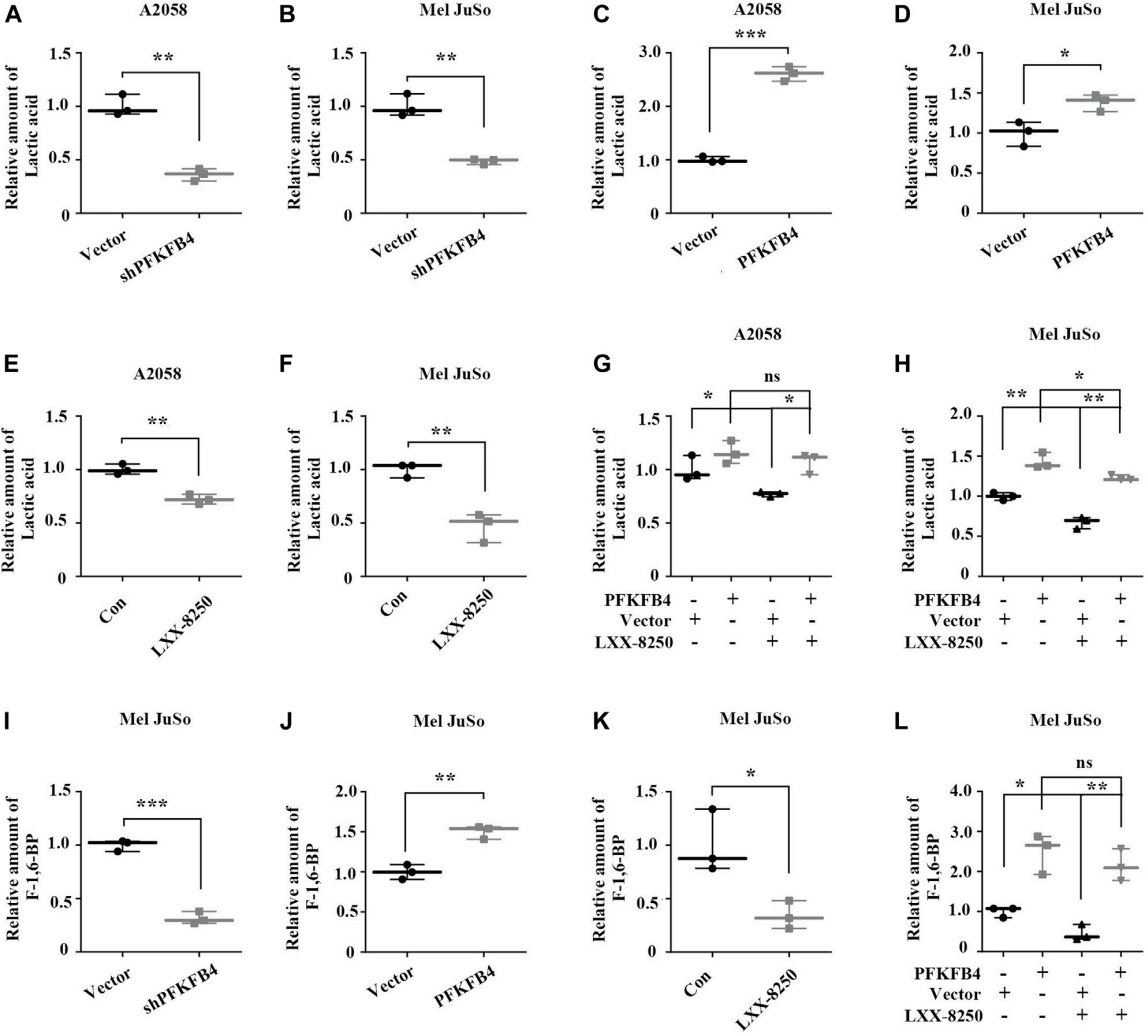
Effects of LXX-8250 and PFKFB4 on lactic acid production. **(A–H)** Lactic acid analyses. The indicated cells were introduced with empty vector or PFKFB4 shRNA (shPFKFB4) **(A,B)**. The indicated cells were introduced with empty vector or PFKFB4 overexpression plasmid **(C,D)**. The indicated cells were treated with or without LXX-8250 14.6 μM, **(E)**; 22.8 µΜ, **(F) (E,F)**. The indicated cells introduced with empty vector or PFKFB4 overexpression plasmid were treated with or without LXX-8250 14.6 μM, **(G)**; 22.8 µΜ, **(H)** for 24 h **(G,H)**. **(I–L)** F-1,6-BP analyses. The indicated cells were introduced with empty vector or PFKFB4 shRNA (shPFKFB4) **(I)**. The indicated cells were introduced with empty vector or PFKFB4 overexpression plasmid **(J)**. The indicated cells were treated with or without LXX-8250 22.8 µΜ, **(K)**. The indicated cells introduced with empty vector or PFKFB4 overexpression plasmid were treated with or without LXX-8250 22.8 µΜ, **(L)** for 24 h. Data represent mean ± SD. *p < 0.05, **p < 0.01, ***p < 0.001.

Since PFKFB4 is an upstream factor of glycolysis and plays a role in glucose metabolism, we examined the effects of PFKFB4 on glycolysis through evaluating the production of lactic acid. The media from PFKFB4-knockdown cells (shPFKFB4) and control cells were applied to lactic acid analyses. Similar to the previous reports, the lactic acid concentration was greatly lower in the media of PFKFB4-knockdown cells than that in the media of control cells (*p* < 0.01, Figures 5A,B), while was much higher in the media of PFKFB4-overexpressing cells (*p* < 0.01, Figures 5C,D). Thus, PFKFB4 facilitates lactic acid production.

Next LXX-8250’s effects on the production of lactic acid were investigated. The lactic acid concentration in the media of the cells treated with LXX-8250 was significantly decreased (*p* < 0.01, Figures 5E,F). When PFKFB4 was overexpressed, the lactic acid concentration in the media of A2058 cells treated with LXX-8250 was similar to that of the control treated cells. The decrease of the lactic acid concentration in the media of PFKFB4-transfected Mel JuSo cells by LXX-8250 treatment was less than that in empty vector transfected cells (Figures 5G,H). Thus PFKFB4-overexpressing cells showed resistance to LXX-8250-suppressed lactic acid production. Since the conversion of F6P to F-1,6-BP catalyzed by PFK1 is the committed step of glycolytic flux, we also examined the effects of PFKFB4 and LXX-8250 treatment on F-1,6-BP production (Figures 5I–L). Similar as the effects on lactic acid production, PFKFB4 expression promotes F-1,6-BP production, and LXX-8250 treatment remarkably inhibited F-1,6-BP production, which was tolerant in PFKFB4-overexpressing cells. On the contrary, essential glycolytic enzymes PGK1 and LDHA were not found to be affected by LXX-8250 treatment (Supplementary Figure S3). These results suggest that LXX-8250 treatment may inhibit glycolysis *via* suppressing PFKFB4 expression.

### LXX-8250 Treatment Retards the Growth of Melanoma *In Vivo*

**FIGURE 6:**
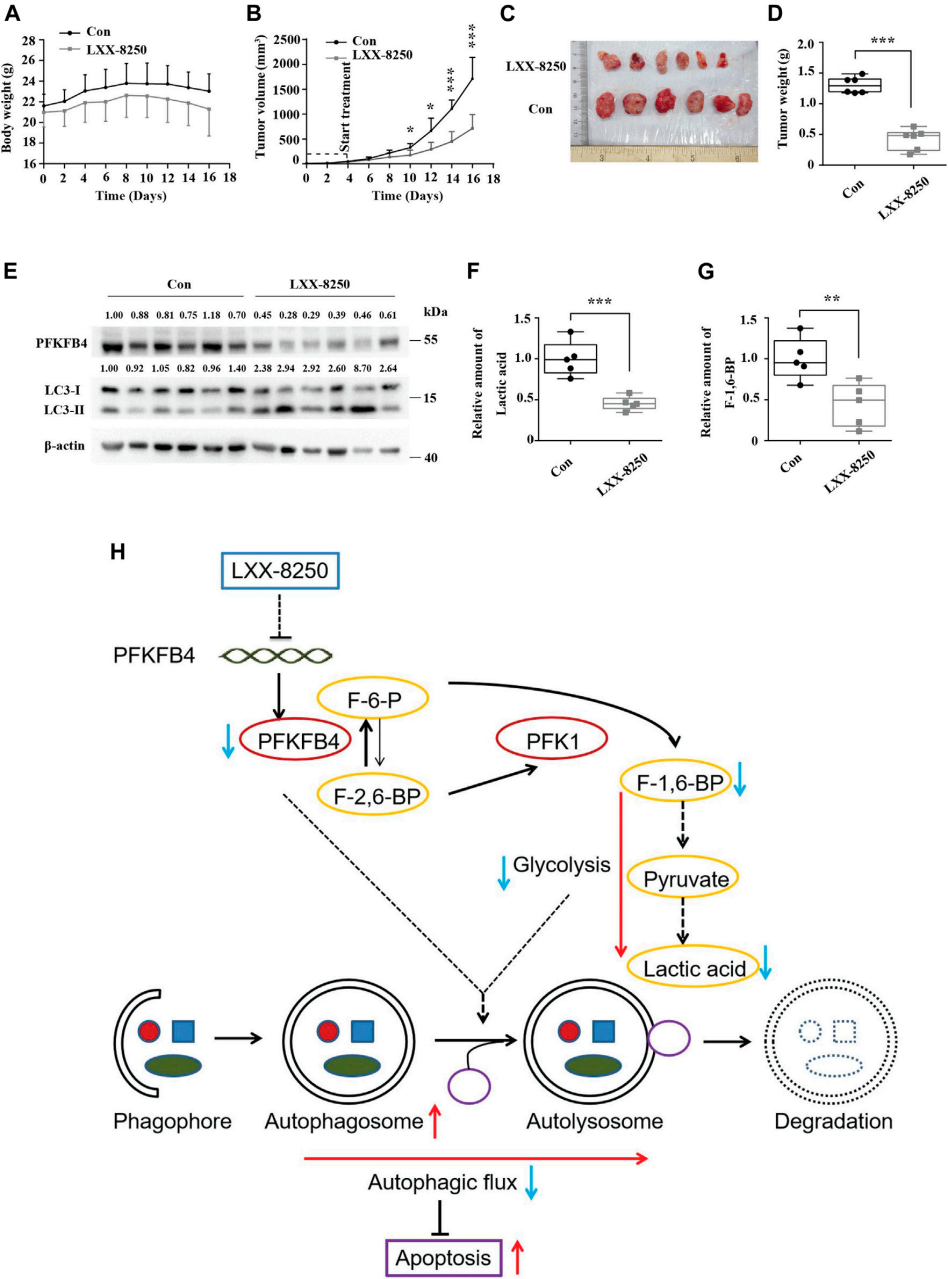
Effects of LXX-8250 treatment on the growth of melanoma xenografts. The mice with A2058 xenografts were treated with LXX-8250 (20 mg/kg), and DMSO was used as a control treatment (*n* = 6). **(A)** Every 2 days body weights of mice were recorded. **(B)** Every 2 days tumor volumes were recorded. **(C)** On day 17, the tumors were obtained. Pictures shown were taken at the time of sacrifice. **(D)** Tumors’ weights were measured. **(E)** Western blot analysis for the tumor tissue lysates. The quantification results of the band density (PFKFB4 and LC3-II) were labeled above the bands. **(F)** Lactic acid analyses for the tumor tissue lysates. **(G)** F-1,6-BP analyses for the tumor tissue lysates. **(H)** Schematic graph representing the working model of LXX-8250. F-6-P, fructose-6-phosphate; F-1,6-BP, fructose-1,6-biphosphate; F-2,6-BP, fructose-2,6-biphosphate. →, promotion; ┤, suppression. Data represent mean ± SD. *p < 0.05, **p < 0.01, ***p < 0.001.

Based on the *in vitro* results, we further explored the possibility that LXX-8250 treatment inhibits the growth of melanoma xenografts in mice. The body weights of the mice treated with LXX-8250 were not changed significantly compared with those of control treatment (Figure 6A). From the 6th day after the beginning of LXX-8250 treatment, the tumor xenograft growth was remarkably inhibited compared with that of the control treatment, and LXX-8250 treatment significantly reduced the tumor volume and weight (*p* < 0.001, Figures 6B–D). Moreover, PFKFB4 expression was decreased and LC3-II expression was increased in the samples of LXX-8250-treated mice compared to those of the control mice (Figure 6E). Additionally, the levels of lactic acid and F-1,6-BP in the xenograft samples of LXX-8250-treated mice were significantly reduced compared with those of the control mice (*p* < 0.01, Figures 6F,G). Collectively these data suggest that LXX-8250 treatment inhibits the growth of melanoma *in vivo via* repressing PFKFB4 expression and glycolysis (Figure 6H).

## Discussion

Melanoma is the leading cause of skin cancer death worldwide. Although immunotherapies and targeted therapies show efficacy in melanoma recently, drug resistance remains an obstacle (Kim and Cohen, 2016; Wang et al., 2018b; Tabolacci et al., 2021). Therefore, novel approaches to melanoma treatment are needed with great urgency.

β-elemene inhibits proliferation and induces apoptosis in a variety of tumors (Zheng et al., 2018; Wu et al., 2019), whereas the IC_50_s for tumor cells are comparatively high, such as glioma (240 µΜ) (Zhu et al., 2013), lung cancer (27.5 μg/ml = 134.6 µΜ) (Liu et al., 2017), ovarian cancer (307.7 µΜ) (Zou et al., 2013), prostate cancer (230–465 µΜ) (Li et al., 2010), gastric cancer (56.03 μg/ml = 274.2 µΜ) (Liu et al., 2011), leukemia (42.19 μg/ml = 206.5 µΜ) (Jiang et al., 2019), and melanoma (468.8 µΜ) (Chen et al., 2011). In addition, no representative clinical study has been conducted to explore the effects of β-elemene on controlling melanoma earlier. Herein we showed that the treatment with a novel β-elemene derivative LXX-8250 induced apoptosis of melanoma cells, and the IC_50_s for 48hr-treatment are 13.5–22.8 µΜ, less than one twentieth of the IC_50_ of β-elemene and one fourth of the IC_50_ of Dacarbazine for melanoma cells (Figure 1 and Supplementary Figure S1).

Autophagy is crucial in cell survival. Inhibition of autophagy impairs nutrient recycling and energy generating, resulting in apoptosis (MaiuriZalckvar et al., 2007). In LXX-8250-treated melanoma cells, LC3-II and P62 expression levels were increased a lot, which was not upregulated further by combination with CQ treatment. Puncta analysis showed that LXX-8250 treatment augmented significantly the amounts of autophagosome but not autolysosome. Therefore LXX-8250 suppressed autophagosome degradation, resulting in inhibition of autophagic flux (Figures 2A–D, Supplementary Figure S2). Furthermore, 3-MA treatment reversed the growth inhibition and PARP cleavage by LXX-8250, which proved that the autophagosome accumulation led by LXX-8250 treatment should be responsible to the apoptosis induction in melanoma cells (Figures 2E–H). Under the treatment with BRAF inhibitors or MEK inhibitors, tumor cells including melanoma finally adapt to drug treatment by upregulation of autophagy (Martin et al., 2017; Mulcahy Levy et al., 2017), suggesting the availability of autophagy inhibitors in combination with targeted therapies. This possibility should be also considered for LXX-8250 in the future.

Next the molecular mechanism of LXX-8250’s antitumor effects should be determined. The glycolysis enzyme PFKFB4 ranked No. 2 dependent on the fold change in the genes downregulated by LXX-8250 treatment (Figure 3A). Moreover, increasing attention has given to the role of Warburg effects in tumor, and β-elemene was reported to be able to downregulate aerobic glycolysis (Pan et al., 2019). Therefore, we focused on the role of PFKFB4 in the effects of LXX-8250 in melanoma, and confirmed the downregulation of PFKFB4 expression by LXX-8250 treatment (Figures 3C–F, Figure 6D).

Analysis of TCGA and GTEx data showed that PFKFB4 was significantly upregulated in melanoma compared with those in normal tissue (Figure 3B). Recently, PFKFB4 has been reported for various biological functions, such as deregulating cell proliferation, autophagy, apoptosis, and metastasis in a variety of tumors (Strohecker et al., 2015; Wang et al., 2018a; Dasgupta et al., 2018; Zhang et al., 2021b; Zhang et al., 2021c; Meng et al., 2021; Truong et al., 2021). Consistent with the previous studies, our results showed that PFKFB4 knockdown inhibited cell proliferation (Figures 4A–F). In addition, the expression levels of LC3-II and P62 were increased in PFKFB4-knockdown cells while decreased in PFKFB4-overexpressing cells (Supplementary Figure S3). Furthermore, PFKFB4 overexpression showed significant resistance to the apoptosis induction as well as upregulation of LC3-II and P62 levels by LXX-8250 treatment (Figures 4G–O). Therefore LXX-8250 works as a novel suppressor of PFKFB4 to inhibit autophagic flux and induce apoptosis in melanoma.

PFKFB4 is an important enzyme of glycolysis, directly related to more glucose consumption and lactic acid production (Guo et al., 2020). Whether PFKFB4 repression by LXX-8250 treatment in melanoma cells leads to glycolysis inhibition attracted our attention. Here we reported that shPFKFB4 transfection reduced the production of lactic acid and F-1,6-BP while PFKFB4 overexpression increased the production of lactic acid and F-1,6-BP in melanoma cells. LXX-8250 treatment decreased the production of lactic acid and F-1,6-BP both *in vitro* and *in vivo*. Furthermore, the melanoma cells with PFKFB4 overexpression were resistant to the reduction of the levels of lactic acid and F-1,6-BP by LXX-8250 treatment (Figure 5). These results indicate that LXX-8250 suppresses PFKFB4 to inhibit glycolysis.

Finally the effects of LXX-8250 treatment were confirmed in melanoma xenograft models. LXX-8250 treatment inhibited the tumor growth significantly *in vivo*, which was associated by LC3-II accumulation and glycolysis suppression (Figure 6). This study provides experimental evidence for a novel approach to melanoma by LXX-8250 treatment. Further studies are needed for the potential clinical application to treat melanoma.

## Conclusion

Our results demonstrated that β-elemene isopropanolamine derivative LXX-8250 induced apoptosis by inhibiting autophagic flux and glycolysis *via* suppression of PFKFB4 expression in melanoma cells (Figure 6H).

## Data Availability

The original contributions presented in the study are included in the article/Supplementary Materials, further inquiries can be directed to the corresponding author.
