# Supplementary material for: β-elemene Isopropanolamine Derivative LXX-8250 Induces Apoptosis Through Impairing Autophagic Flux via PFKFB4 Repression in Melanoma Cells
Source: Front Pharmacol. 2022 Aug 10;13:900973. doi: 10.3389/fphar.2022.900973 (PMC9399853; doi:10.3389/fphar.2022.900973)
Supplement: Supplementary file 1 [file DataSheet1.docx]

**β-elemene isopropanolamine derivative LXX-8250 induces apoptosis through impairing autophagic flux via PFKFB4 repression in melanoma cells**

Sajid Jalal^1^, Ting Zhang^1^, Jia Deng^1^, Jie Wang^1^, Ting Xu^2^, Tianhua Zhang^1^, Chuanxin Zhai^1^, Ruqiang Yuan^3^, Hongming Teng^1^, Lin Huang^1^


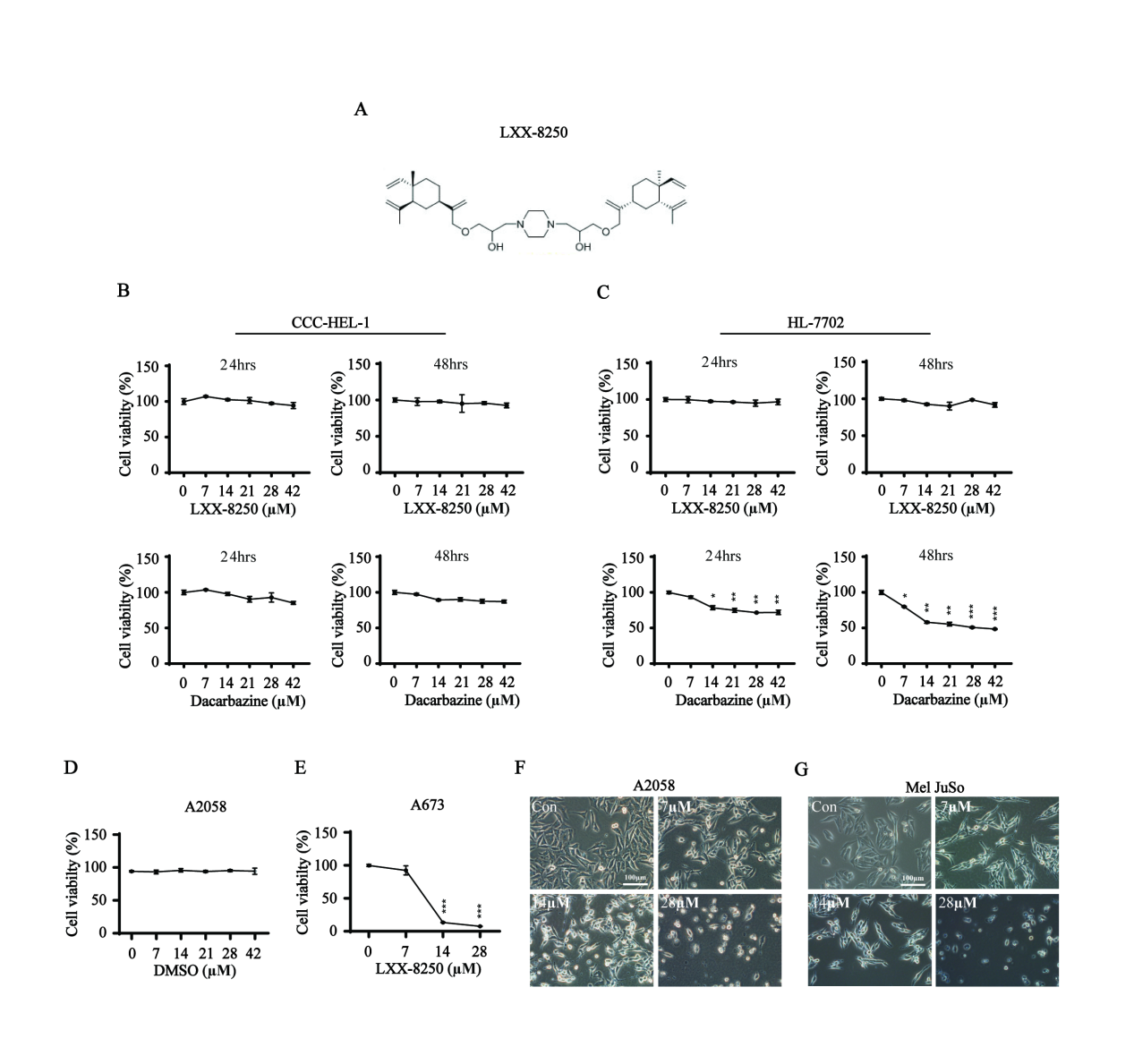
Supplementary Fig. 1. The effects of LXX-8250 on Melanoma cells. (A) Chemical structure of LXX-8250 (3,3′-(Piperazine-1,4-diyl)-bis(1-((2-((1R,3S,4S)-4-methyl- 3-(prop-1-en-2-yl)-4-vinylcyclohexyl)allyl)oxy)propan-2-ol)). (B-E) The indicated cells treated with indicated concentration of LXX-8250, Dacarbazine, or DMSO for 48 hrs. Cytotoxicity was analyzed with cell viability assay. (F-G) Morphological changes of LXX-8250 treated cells with a scale bar of 100µm. Data represent mean ± SD. * *P*<0.05, ** *P*<0.01, *** *P*<0.001.


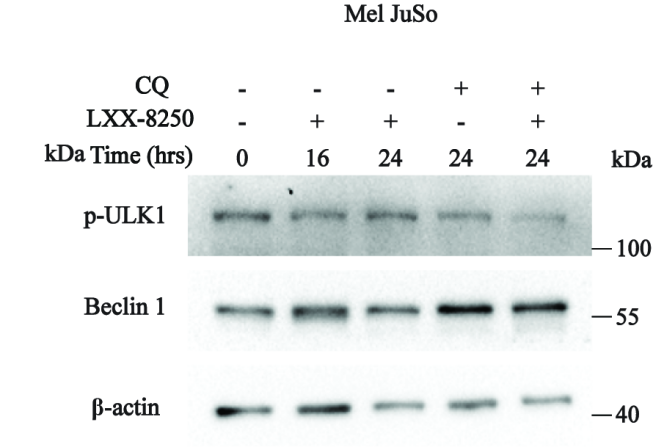


Supplementary Fig. 2. The effects of LXX-8250 on autophagic factors. Western blot analysis for the cells with the indicated treatment at the indicated time. LXX-8250 (22.8µΜ), CQ, 10μM. The quantification results of the band density were labeled above the bands.


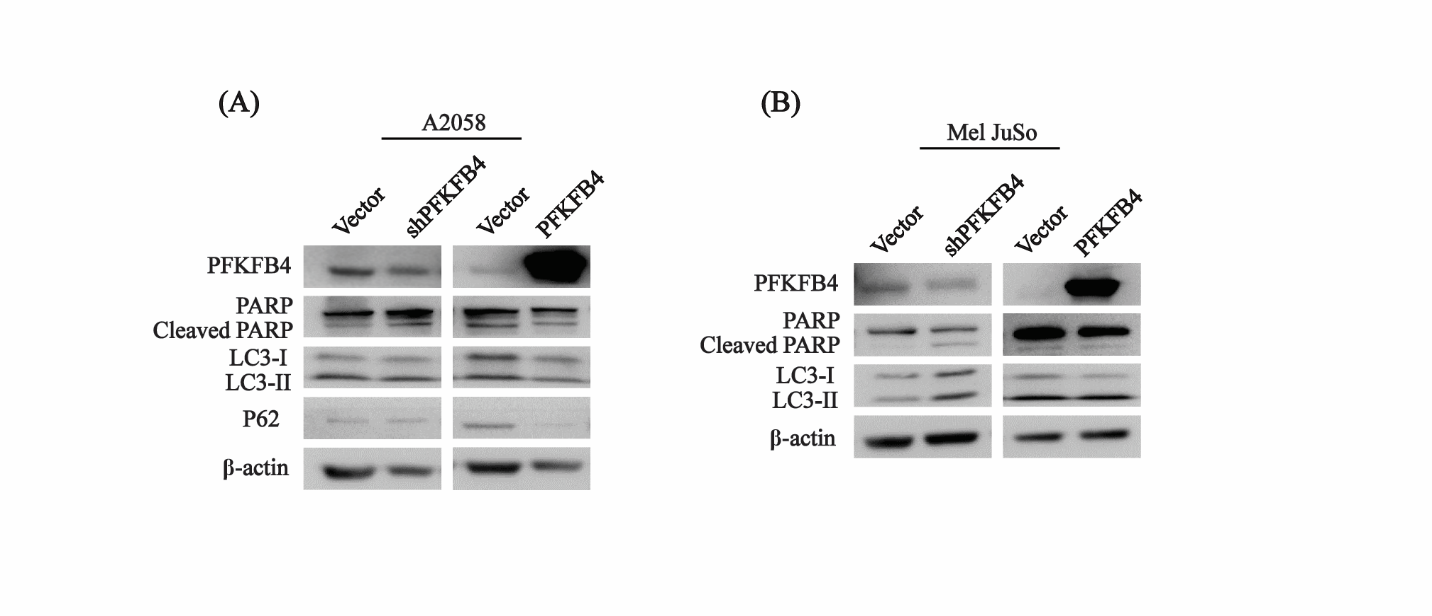
Supplementary Fig. 3. The effects of PFKFB4 on autophagy and apoptosis markers detected with Western blot analysis in A2058 cells (A) and Mel Juso cells (B).


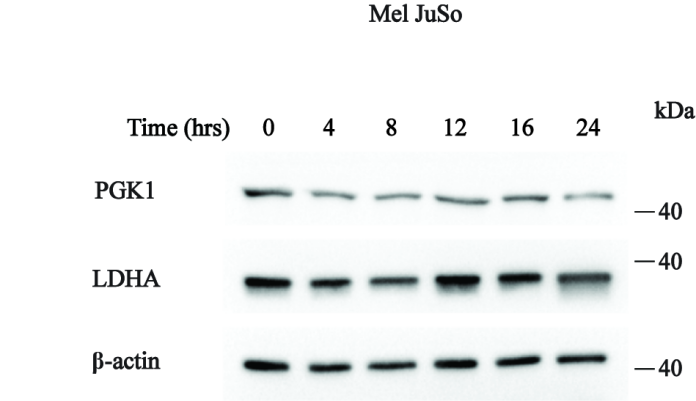


Supplementary Fig. 4. The effects of LXX-8250 on glycolytic enzymes. Western blot analysis for the cells treated with LXX-8250 (22.8µΜ) for the indicated time. The quantification results of the band density were labeled above the bands.
